# Supplementary material for: Genome-wide functional screens enable the prediction of high activity CRISPR-Cas9 and -Cas12a guides in Yarrowia lipolytica
Source: Nat Commun. 2022 Feb 17;13:922. doi: 10.1038/s41467-022-28540-0 (PMC8854577; doi:10.1038/s41467-022-28540-0)
Supplement: Supplementary file 1 — Supplementary Information file [file 41467_2022_28540_MOESM1_ESM.pdf]

Supplementary Information

Genome-wide functional screens enable the prediction of high activity CRISPR-Cas9 and -Cas12a guides in *Yarrowia lipolytica*

Dipankar Baisya[1,#], Adithya Ramesh[2,#] , Cory Schwartz[2,†], Stefano Lonardi[1,3*], and Ian Wheeldon[2,3,4*]

1. Department of Computer Science, University of California, Riverside, CA 92521
2. Department of Chemical and Environmental Engineering, University of California, Riverside, CA 92521
3. Integrative Institute for Genome Biology, University of California, Riverside, CA 92521
4. Center for Industrial Biotechnology, University of California, Riverside, CA 92521

# These authors contributed equally

† Current address: iBio Inc., San Diego, CA

* Corresponding authors: wheeldon@ucr.edu, stelo@cs.ucr.edu

Supplementary Figure 1: Design and validation of Cas12a sgRNA library for *Y. lipolytica.*
Supplementary Figure 2: Replicate correlations at day 4 of the growth screen for Cas12a experiments.
Supplementary Table 1: All replicate correlations for the Cas9 and Cas12a genome-wide growth screens.
Supplementary Table 2: The twelve layers of DeepGuides' convolutional auto-encoder.
Supplementary Table 3: The eleven layers in the second network of DeepGuide.
Supplementary Table 4: Ablation analysis on Cas12a dataset.
Supplementary Table 5: Ablation analysis on Cas9 dataset.
Supplementary Figure 3: Genes used to validate DeepGuide and the observed phenotype of null mutants.
Supplementary Figure 4: Clustering of high and poor activity guides used for validation.
Supplementary Figure 5: AUROC curves for all tools on Cas9 and Cas12a datasets.
Supplementary Figure 6: Training and validation loss for DeepGuide with and without pre-training.
Supplementary Figure 7: Evaluation of DeepGuide's ability to predict guide activity in other species.
Supplementary Table 6: Strains of yeast used in this study.
Supplementary Table 7: Plasmids used or generated in this study.
Supplementary Table 8: Primers used in this study.
Supplementary Table 9: Transformation efficiencies achieved in the control and sample screens.
Supplementary Table 10: Primers used for NGS fragment amplification.
Supplementary Figure 8: Schematic and sequence information of Cas9 and Cas12a amplicons for NGS.

Supplementary Table 11: Parameters for bioinformatics tools used in analysis of NGS reads.
Supplementary Table 12: Correlation of SRA files names to demultiplexing information.

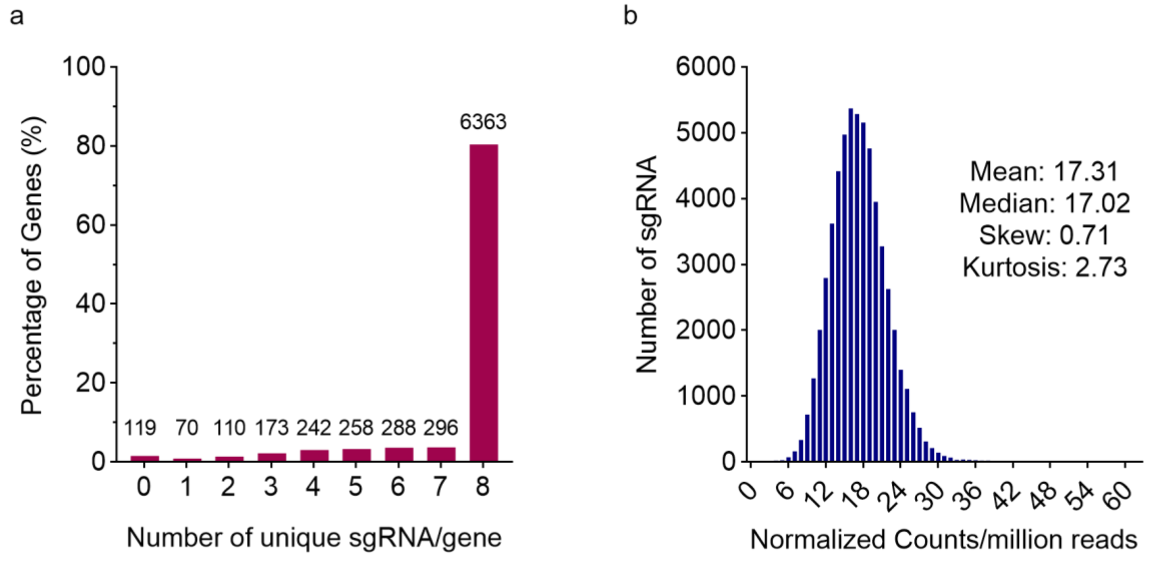

Supplementary Figure 1. Design and validation of Cas12a and Cas9 sgRNA library for *Y. lipolytica* PO1f. (a) An 8-fold redundant sgRNA library was designed to target 7,919 protein coding genes in the *Y. lipolytica* CLIB89 strain, the parent strain of PO1f. Coding sequences were confirmed to be present in the PO1f genome sequence. Over 80% of the genes had 8 sgRNAs and over 91% of the genes had at least 5 sgRNAs. (b) A library consisting of 58,421 sgRNAs was synthesized by Agilent, cloned in-house and characterized by next generation sequencing. The library exhibited a tight normal distribution with nearly equal mean and median signifying minimal skew. The average representation of sgRNAs was ~100-fold (at 5.84 million reads which is 100 times the library size, we can calculate the mean representation of sgRNAs to be 5.84*17.31 = 101.09). **Note**: The Cas9 library design was previously reported in ref. 1 (see Figure S1). Additional details of this library are also provided in the materials and methods section of this manuscript.

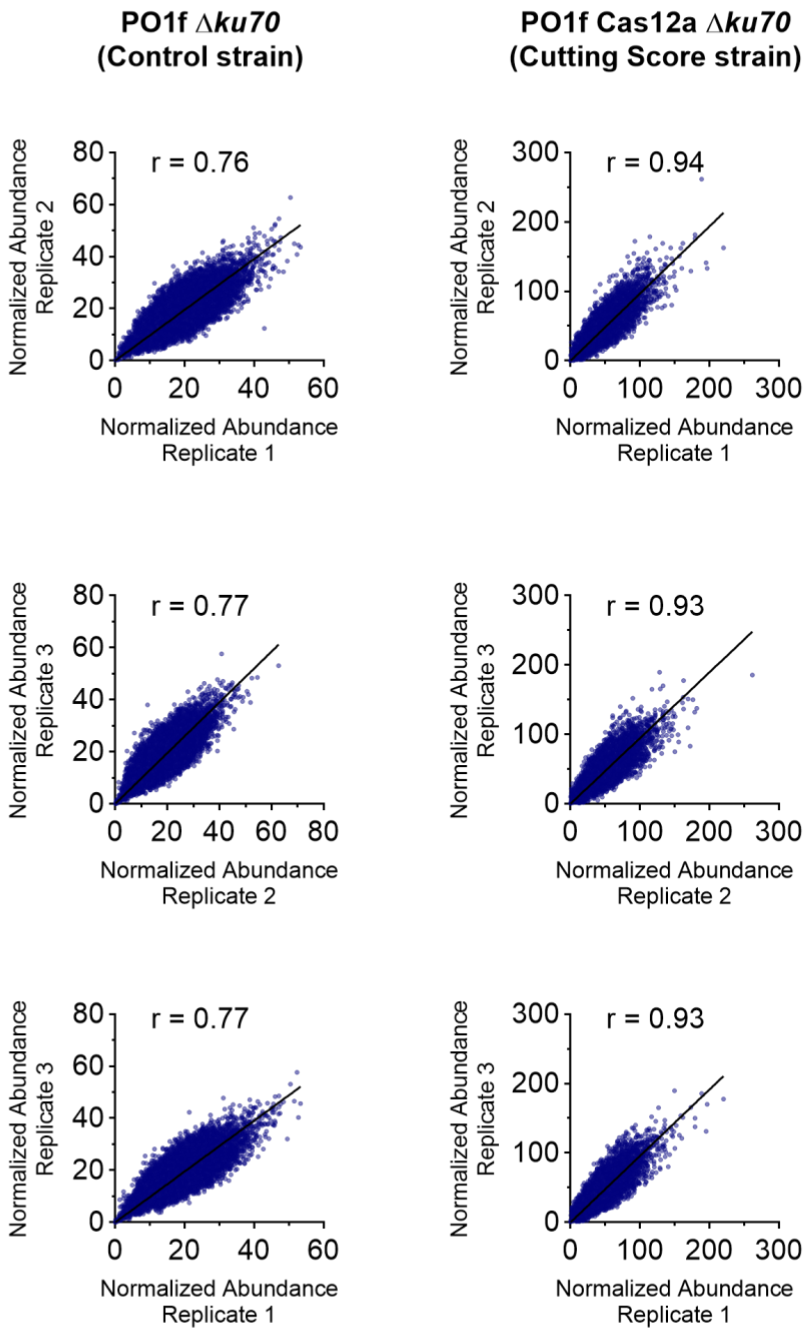

Supplementary Figure 2. Replicate correlation graphs at Day 4 of the growth screen for Cas12a experiments. The column on the left shows pairwise correlations for the control strain while the column on the right shows the same for sample strain.

Supplementary Table 1. Replicate correlations for the genome-wide growth screens in *Y. lipolytica* with the Cas9 and Cas12a endonucleases. Cas9 data was previously reported in ref. [1] Note: Work conducted in ref. 1 uses PO1f with functional KU70 as the control strain.

Strain	Time point	Comparison	Pearson
PO1f *ku70*	Day 2	1 v. 2	0.765
		1 v. 3	0.775
		2 v. 3	0.738
	Day 4	1 v. 2	0.756
		1 v. 3	0.772
		2 v. 3	0.762
	Day 6	1 v. 2	0.797
		1 v. 3	0.768
		2 v. 3	0.799
PO1f Cas12a *ku70*	Day 2	1 v. 2	0.902
		1 v. 3	0.925
		2 v. 3	0.892
	Day 4	1 v. 2	0.936
		1 v. 3	0.933
		2 v. 3	0.927
	Day 6	1 v. 2	0.918
		1 v. 3	0.915
		2 v. 3	0.905

Strain	Time point	Comparison	Pearson
PO1f	Day 2	1 v. 2	0.988
		1 v. 3	0.982
		2 v. 3	0.980
	Day 4	1 v. 2	0.829
		1 v. 3	0.827
		2 v. 3	0.858
	Day 6	1 v. 2	0.818
		1 v. 3	0.829
		2 v. 3	0.855
PO1f Cas9 *ku70*	Day 2	1 v. 2	0.972
		1 v. 3	0.976
		2 v. 3	0.972
	Day 4	1 v. 2	0.886
		1 v. 3	0.891
		2 v. 3	0.973
	Day 6	1 v. 2	0.877
		1 v. 3	0.875
		2 v. 3	0.968

Supplementary Table 2. The twelve layers in the convolutional auto-encoder (first network in DeepGuide); the autoencoder is composed by an encoder (layers 1-6) and a decoder (layers 7-12).

**CAE (1st network)**	**Layer #**	**Layer type**
**Encoder**	1	Convolution
	2	Batch Normalization
	3	Max Pooling
	4	Convolution
	5	Batch Normalization
	6	Average Pooling
**Decoder**	7	Up Sampling
	8	Batch Normalization
	9	Convolution
	10	Up Sampling
	11	Batch Normalization
	12	Convolution

Supplementary Table 3. The eleven layers in the second network in DeepGuide, composed of an encoder (layers 1-6) and a fully connected network (layers 7-11).

2nd network	Layer #	Layer type
**Encoder**	1	Convolution
	2	Batch Normalization
	3	Max Pooling
	4	Convolution
	5	Batch Normalization
	6	Average Pooling
**Fully connected network**	7	Flatten
	8	Fully connected
	9	Fully connected
	10	Fully connected
	11	Multiplication

Supplementary Table 4. Ablation analysis on Cas12a dataset; green row (row 1) show the performance of the encoder (followed by a flatten layer) using random weights (no pre-training or backpropagation); purple row (row 2) show the performance of the encoder (followed by a flatten layer) using random weights and then performing back-propagation only on the flatten layer; blue rows (3-7) show the performance after pre-training the encoder and then running back-propagation only layers downstream of the encoder; pink rows (8-12) show the performance after pre-training and then running back-propagation on the whole network (including the encoder); correlation coefficients in bold corresponds to the best performance; fc = fully connected layer; pool = pooling layer; flatten = flatten layer; mult = multiplication layer (see Table S5 for the list of layers)

Cas12a	Layers	Spearman	Pearson
No pre-training (random weights), **no** back-propagation	encoder⇨flatten$_{7}$	0.060	0.070
No pre-training (random weights), followed by back-propagation **only** on the flatten layer	encoder⇨flatten$_{7}$	0.451	0.455
Pre-training of the encoder followed by back-propagation **only** the layers downstream of the encoder (flatten$_{7}$⇨…)	encoder⇨flatten$_{7}$	0.521	0.532
	encoder⇨flatten$_{7}$⇨fc$_{8}$	**0.527**	**0.534**
	encoder⇨flatten$_{7}$⇨fc$_{8}$⇨fc$_{9}$	0.505	0.517
	encoder⇨flatten$_{7}$⇨fc$_{8}$⇨fc$_{9}$⇨fc$_{10}$	0.501	0.514
	encoder⇨flatten$_{7}$⇨fc$_{8}$⇨fc$_{9}$⇨fc$_{10}$⇨mult$_{11}$	0.501	0.514
Pre-training of the encoder followed by back-propagation on the entire network	encoder⇨flatten$_{7}$	0.637	0.641
	encoder⇨flatten$_{7}$⇨fc$_{8}$	0.649	0.658
	encoder⇨flatten$_{7}$⇨fc$_{8}$⇨fc$_{9}$	**0.653**	**0.660**
	encoder⇨flatten$_{7}$⇨fc$_{8}$⇨fc$_{9}$⇨fc$_{10}$	0.653	0.660
	encoder⇨flatten$_{7}$⇨fc$_{8}$⇨fc$_{9}$⇨fc$_{10}$⇨mult$_{11}$	0.653	0.660

Supplementary Table 5. Ablation analysis on Cas9 dataset; dataset; green row (row 1) show the performance of the encoder (followed by a flatten layer) using random weights (no pre-training or backpropagation); purple row (row 2) show the performance of the encoder (followed by a flatten layer) using random weights and then performing back-propagation only on the flatten layer; blue rows (3-7) show the performance after pre-training the encoder and then running back-propagation only layers downstream of the encoder; pink rows (8-12) show the performance after pre-training and then running back-propagation on the whole network (including the encoder); correlation coefficients in bold corresponds to the best performance; fc = fully connected layer; pool = pooling layer; flatten = flatten layer; mult = multiplication layer (see Table S5 for the list of layers)

Cas9	Layers	Spearman r	Pearson r
No pre-training (random weights), **no** back-propagation	encoder⇨flatten$_7$	0.004	0.003
No pre-training (random weights), followed by back-propagation **only** on the flatten layer	encoder⇨flatten$_7$	0.291	0.312
Pre-training of the encoder followed by back-propagation **only** the layers downstream of the encoder (flatten$_7$⇨…)	encoder⇨flatten$_7$	0.316	0.353
	encoder⇨flatten$_7$⇨fc$_8$	0.273	0.310
	encoder⇨flatten$_7$⇨fc$_8$⇨fc$_9$	0.261	0.291
	encoder⇨flatten$_7$⇨fc$_8$⇨fc$_9$⇨fc$_{10}$	0.269	0.305
	encoder⇨flatten$_7$⇨fc$_8$⇨fc$_9$⇨fc$_{10}$⇨mult$_{11}$	**0.345**	**0.388**
Pre-training of the encoder followed by back-propagation on the entire network	encoder⇨flatten$_7$	0.347	0.409
	encoder⇨flatten$_7$⇨fc$_8$	0.364	0.424
	encoder⇨flatten$_7$⇨fc$_8$⇨fc$_9$	0.357	0.414
	encoder⇨flatten$_7$⇨fc$_8$⇨fc$_9$⇨fc$_{10}$	0.357	0.414
	encoder⇨flatten$_7$⇨fc$_8$⇨fc$_9$⇨fc$_{10}$⇨mult$_{11}$	**0.431**	**0.501**

Gene Name	Function	Observed phenotype of null
MGA1	Heat shock factor & pseudohyphal growth	Smooth colonies
RAS2	GTP-binding protein, regulates filamentous growth	Smooth colonies
CAN1	Arginine permease	Canavanine resistance
MFE1	β-oxidation of long chain fatty acids	Oleic acid metabolism muted

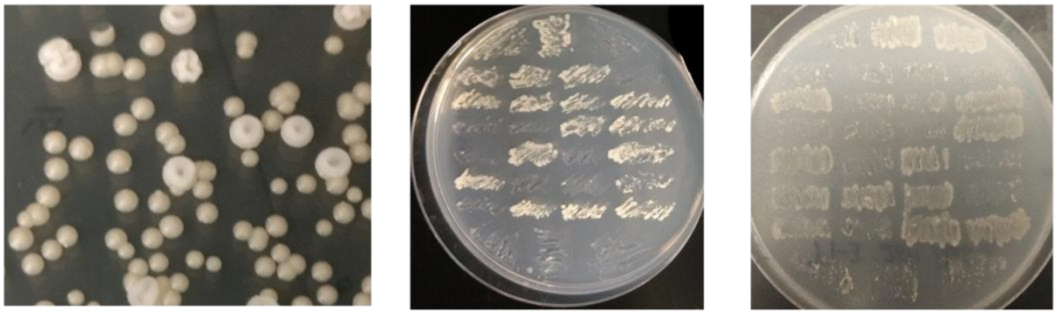

ΔRAS2 & ΔMGA1   *ΔCAN1*   *ΔMFE1*

Supplementary Figure 3. Genes selected for experimental validation of DeepGuide and the observed phenotype of the null mutants. MGA1 and RAS2 are implicated in the pseudohyphal and filamentous growth, and their null mutants show smooth colonies as shown in the picture on the left. CAN1 disruption confers resistance to L-Canavanine which is a toxic analog of Arginine. This leads to growth on plates supplemented with canavanine, as shown in the middle picture. MFE1 disruption renders *Y. lipolytica* unable to utilize oleic acid as a carbon source, and null mutants do not grow on plates with oleic acid as the sole carbon source as shown in the right-most picture.

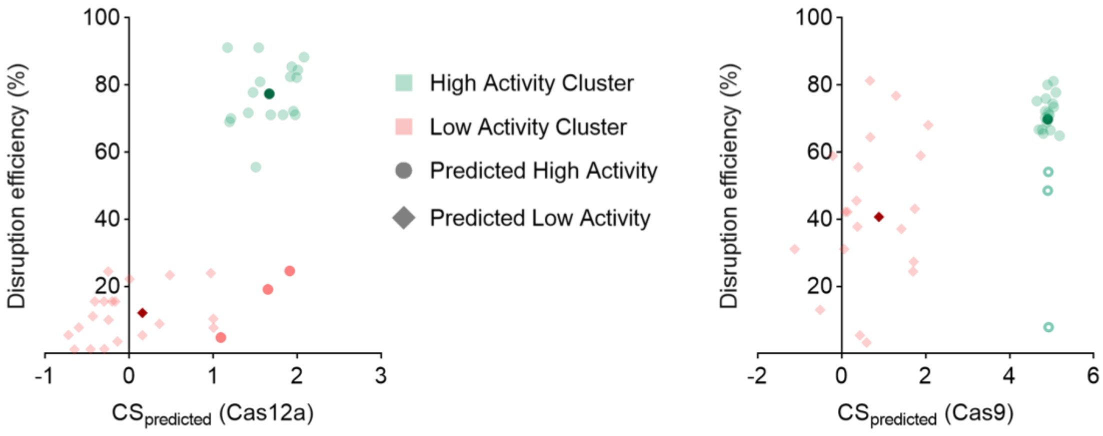

Supplementary Figure 4. Clustering of high and poor activity guides used to validate DeepGuide. Predicted CS values and experimental disruption efficiencies for both the Cas12a and Cas9 were plotted on an XY scatter plot and a gaussian mixture model was used to cluster the sgRNA into two clusters (high and low activity). The high activity clusters are indicated in green, while the low activity clusters are indicated in red. Dark green and red points correspond to cluster centroids. Data point shape indicates whether the guide was predicted to be of high or low activity (circles are high activity, diamonds are low activity). Three predicted high activity guides cluster with low activity guides for Cas12a. For Cas9, three guides in the high activity cluster have a significantly higher euclidean distance from the cluster centroid and appear to be outliers (marked with empty circles).

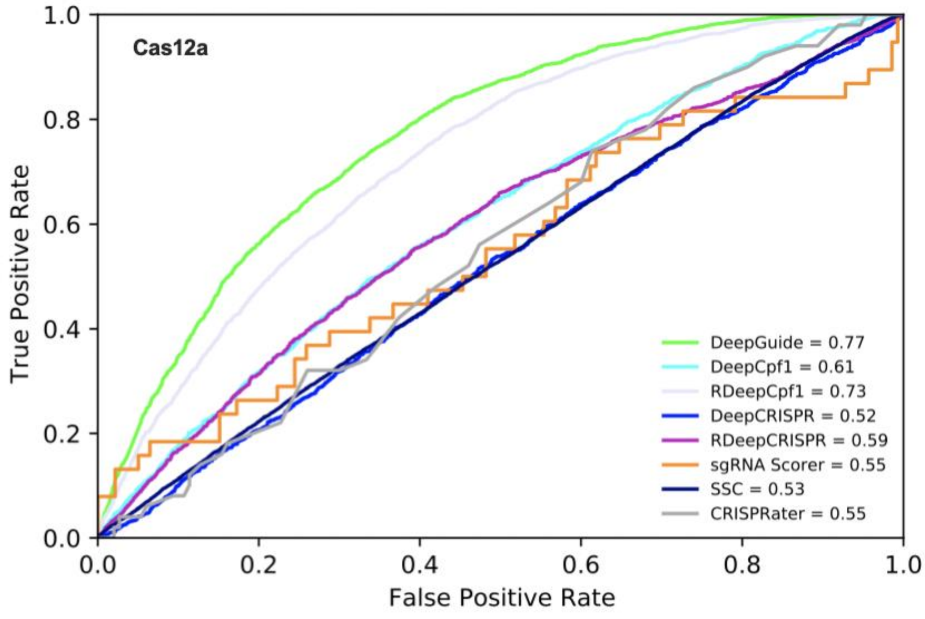

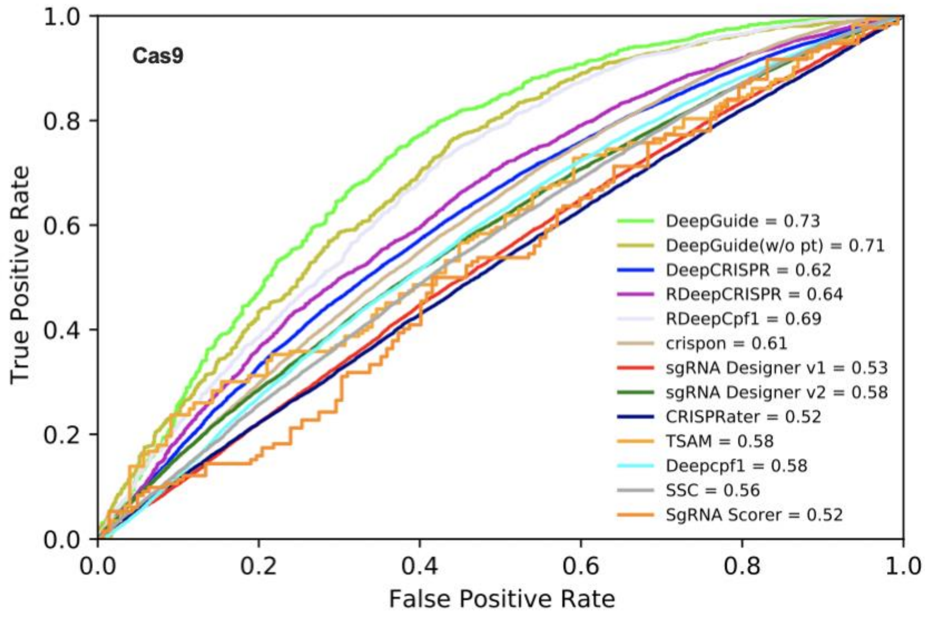

Supplementary Figure 5. ROC plots and AUROC values for DeepGuide, DeepCpf1 (original and retrained), DeepCRISPR (original and retrained), sgRNA Scorer, SSC, and CRISPRater for the prediction of sgRNA activity on the Cas12a dataset (left) and the Cas9 dataset (right). DeepGuide had higher AUROC values than all other guide activity prediction algorithms. Guides with CS > 1.67 for Cas12a and CS > 4.91 for Cas9 were classified as active, and guides with a CS value below this threshold were classified as inactive. DeepGuide (w/o pt) indicates that no pre-training was carried out.

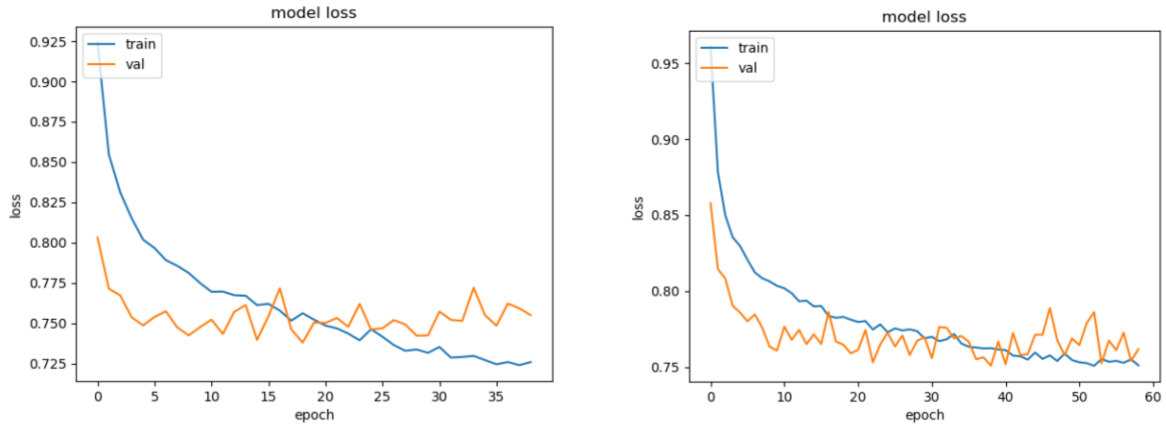

Supplementary Figure 6. Training and validation loss for DeepGuide without pre-training (left) and with pre-training (right) as a function of the number of training epochs. These curves show that pre-training improves the architecture's generalization.

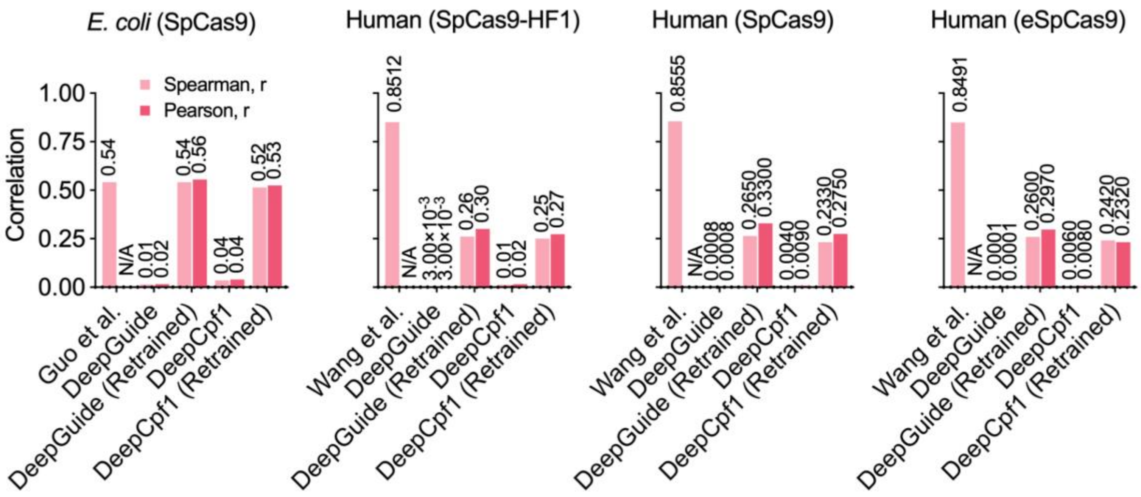

Supplementary Figure 7. Evaluation of DeepGuide's ability to predict guide activity in other species. DeepGuide was tested on four non-Yarrowia datasets, including a CRISPR-Cas9 activity profile in *E. coli* [2] and three CRISPR-Cas9 datasets in mammalian cell lines [3]. These datasets were selected from the 44 publicly available sets listed in ref. 4, because they were the only ones having a size comparable to our *Y. lipolytica* datasets (*i.e.*, they contained at least 30,000 data points; see Figure 4 of main text, DeepGuide requires at least this many data points for high accuracy predictions). DeepGuide, before and after retraining, was compared to DeepCpf1 [5] (also before and after retraining) on all four datasets, as well as to the method originally developed for the respective datasets. DeepCpf1 was chosen because of its strong performance on our *Y. lipolytica* datasets. The data show that (i) retraining is necessary for DeepGuide and DeepCpf1 to achieve a reasonable predictive performance, (ii) when retrained, DeepGuide achieves a slightly higher predictive performance than DeepCpf1. We note that the Spearman coefficient reported on the *E. coli* dataset using the method proposed in ref. 2, which is based on gradient boosting regression trees, was 0.542. This matches the performance of DeepGuide, showing that our method is able to capture CRISPR-Cas9 activity in *E. coli*. DeepGuide was not able to capture guide activity measured in mammalian cell lines, thus demonstrating the importance of architecture optimization for broad cross-species prediction abilities.

Supplementary Table 6. Yeast strains used in this study.

**Yeast strain genotype**	**Phenotype**
PO1f (MatA, *leu2-270*, *ura3-302*, *xpr2-322*, *axp-2*)	Wild type strain
PO1f *Δku70*	PO1f with disrupted KU70, which facilitates the non-homologous end joining DNA repair pathway
PO1f UAS1B8-TEF(136)-Cas9 -CycT::A08	PO1f expressing *Y. lipolytica* codon optimized Cas9 gene at the A08 locus
PO1f UAS1B8-TEF(136)-LbCas12a -CycT::A08	PO1f expressing *Y. lipolytica* codon optimized LbCas12a gene at the A08 locus
PO1f *Δku70* UAS1B8-TEF(136)-Cas9 -CycT::A08	KU70 disrupted in Cas9 integrated PO1f strain
PO1f *Δku70* UAS1B8-TEF(136)-LbCas12a -CycT::A08	KU70 disrupted in LbCas12a integrated PO1f strain

Supplementary Table 7. Plasmids used for genome wide CRISPR screens.

Plasmid name	Reference	Function
pCpf1_yl	[6]	Plasmid for CRISPR-LbCas12a based gene editing in *Y. lipolytica*
pCRISPRyl	[7]	Plasmid for CRISPR-Cas9 based gene editing in *Y. lipolytica*
pLbCas12ayl	This study	Plasmid for CRISPR-LbCas12a based gene editing in *Y. lipolytic*a. sgRNA is flanked on either end by the direct repeat, to allow sgRNAs to end in T residues without being construed as part of the PolyT terminator
pHR_A08_hrGFP (Addgene #84615)	This study	Plasmid containing homology arms for integration of hrGFP into the A08 locus
pHR_A08_LbCas12a	This study	Plasmid containing homology arms for integration of LbCas12a into the A08 locus
pHR_A08_Cas9	[1]	Plasmid containing homology arms for integration of Cas9 into the A08 locus
pLbCas12ayl-GW	This study	Vector containing sgRNA expression cassette for cloning Cas12a sgRNA library. (Does not contain Cas12a expression cassette)
pCas9yl-GW	[1]	Vector containing sgRNA expression cassette for cloning Cas9 sgRNA library. (Does not contain Cas9 expression cassette)
pCRISPRyl_KU70	This study	CRISPR plasmid for the disruption of KU70

Supplementary Table 8. Sequences of primers used in this study.

Primer name	Primer Sequence
ExtraDR-F	CGGCGCAAATTTCTACTAAGTGTAGACTAGTAATTTCTACTAAGTGTAGATTTTTTACGTCTAAGAAACCATTATT
ExtraDR-R	AATAATGGTTTCTTAGACGTAAAAAATCTACACTTAGTAGAAATTACTAGTCTACACTTAGTAGAAATTTGCGCCG
Cpf1-Int-F	TGCCTGGAGCCGAGTACGGCATTGATTACTAGTCCGGGTTCGAAGGTACCAAG
Cpf1-Int-R	TTAGGCTGGGTCTCGAGAGCAAAGAAGCCTAGGGCAAATTAAAGCCTTCGAGCG
BRIDGE-F	CTAAATTTGATGAAAGGGGGATCCCCCGGGTGGCGTAATCATGGTCATAGCTGTTTCCTG
BRIDGE-R	CAGGAAACAGCTATGACCATGATTACGCCACCCGGGGGATCCCCCTTTCATCAAATTTAG
A08-Seq-F	AGCCGAGTACGGCATTGAT
A08-Seq-R	TCAATGTAGCCTCCTCCAACC
Tef_Seq-F	GTTGGGACTTTAGCCAAG
Lb1-R	CTTCTGCTTGGTCTTCTGGTTG
Lb2-F	AACCTGTACAACCAGAAGACCAAG
Lb3-F	AAGGAGACCAACCGAGACGAG
Lb4-F	AACCTGCACACCATGTACTTCAAG
Lb5-F	CCAGATCACCAACAAGTTCGAGTC
M13-F	GTAAAACGACGGCCAGT
InversePCR-F	TTTTTTACGTCTAAGAAACCATTATTATCATGACATTAACCT
InversePCR-R	TGCGCCGACCCGGAATCGAACCGGGGGCCC
OLS-F	GTTTAGTGGTAAAATCCATCGTTGCCATCG
OLS-R	GATACGCCTATTTTTATAGGTTAATGTCATG
qPCR-GW-F	TTATGAACTGAAAGTTGATGGC
qPCR-GW-R	TCACACAGGAAACAGCTATG
Cas9-RAS2-1	TTCGATTCCGGGTCGGCGCACGCGGTCACTCCCCGCTCGTGTTTTAGAGCTAGAAATAGC
Cas9-RAS2-2	TTCGATTCCGGGTCGGCGCACTCCACCAGTGGAGCCAACCGTTTTAGAGCTAGAAATAGC
Cas9-RAS2-3	TTCGATTCCGGGTCGGCGCAACCTCCTGCAGCACCTCCAAGTTTTAGAGCTAGAAATAGC
Cas9-RAS2-4	TTCGATTCCGGGTCGGCGCAGACTCTCAATGCTCCACCAGGTTTTAGAGCTAGAAATAGC
Cas9-RAS2-5	TTCGATTCCGGGTCGGCGCAGATGTCGTAAACCAGAAGATGTTTTAGAGCTAGAAATAGC
Cas9-RAS2-6	TTCGATTCCGGGTCGGCGCAAATCTAGGGCCTCCAAAGACGTTTTAGAGCTAGAAATAGC
Cas9-RAS2-7	TTCGATTCCGGGTCGGCGCATCCCGTTCCTGTGGTTAGTAGTTTTAGAGCTAGAAATAGC
Cas9-RAS2-8	TTCGATTCCGGGTCGGCGCATGTTGGAGTCGACCTGGAAGGTTTTAGAGCTAGAAATAGC

Cas9-RAS2-9	TTCGATTCCGGGTCGGCGCAAAGCTGTGGGTGCACTGGTCG TTTTAGAGCTAGAAATAGC
Cas9-RAS2-10	TTCGATTCCGGGTCGGCGCAGGAACCAGAGGACTAAGCTG GTTTTAGAGCTAGAAATAGC
Cas9-MGA1-1	TTCGATTCCGGGTCGGCGCACTGTTGCGCGGCCTGGGTCGG TTTTAGAGCTAGAAATAGC
Cas9-MGA1-2	TTCGATTCCGGGTCGGCGCAACTGGCCAAGGAGCCTGCTG GTTTTAGAGCTAGAAATAGC
Cas9-MGA1-3	TTCGATTCCGGGTCGGCGCATTGCGGCAGAGGCATGGTTTG TTTTAGAGCTAGAAATAGC
Cas9-MGA1-4	TTCGATTCCGGGTCGGCGCACAGAGGCATGGTTTCGGCGC GTTTTAGAGCTAGAAATAGC
Cas9-MGA1-5	TTCGATTCCGGGTCGGCGCAGCCCGGCGAGGAGTTCTCCA GTTTTAGAGCTAGAAATAGC
Cas9-MGA1-6	TTCGATTCCGGGTCGGCGCAAAGACGGAGTTTGTGGGTGG GTTTTAGAGCTAGAAATAGC
Cas9-MGA1-7	TTCGATTCCGGGTCGGCGCAAGAGAGACAGTGTGCCCTTG GTTTTAGAGCTAGAAATAGC
Cas9-MGA1-8	TTCGATTCCGGGTCGGCGCAGTAGGGGGCGCCTGTCCGTCG TTTTAGAGCTAGAAATAGC
Cas9-MGA1-9	TTCGATTCCGGGTCGGCGCAGAGTGTGGTGGCGGAGTAGA GTTTTAGAGCTAGAAATAGC
Cas9-MGA1-10	TTCGATTCCGGGTCGGCGCATGCGCGGCCTGGGTCGTGGG GTTTTAGAGCTAGAAATAGC
Cas9-CAN1-1	TTCGATTCCGGGTCGGCGCATCAAACGATTACCCACCCTCG TTTTAGAGCTAGAAATAGC
Cas9-CAN1-2	TTCGATTCCGGGTCGGCGCATTACCCACCCTCCGGGACTGG TTTTAGAGCTAGAAATAGC
Cas9-CAN1-3	TTCGATTCCGGGTCGGCGCACCACATCCACATCAACCACAG TTTTAGAGCTAGAAATAGC
Cas9-CAN1-4	TTCGATTCCGGGTCGGCGCACATCAACCACACGGCCCACTG TTTTAGAGCTAGAAATAGC
Cas9-CAN1-5	TTCGATTCCGGGTCGGCGCACACCAGTGGCCACGACCTGG GTTTTAGAGCTAGAAATAGC
Cas9-CAN1-6	TTCGATTCCGGGTCGGCGCAAGTGGGCCGTGTGGTTGATGG TTTTAGAGCTAGAAATAGC
Cas9-CAN1-7	TTCGATTCCGGGTCGGCGCACCGTGTGGTTGATGTGGATGG TTTTAGAGCTAGAAATAGC
Cas9-CAN1-8	TTCGATTCCGGGTCGGCGCAGTGGATGTGGGCCTCAGTCCG TTTTAGAGCTAGAAATAGC
Cas9-CAN1-9	TTCGATTCCGGGTCGGCGCAGATGTGGGCCTCAGTCCCGGG TTTTAGAGCTAGAAATAGC
Cas9-CAN1-10	TTCGATTCCGGGTCGGCGCATGGGCCTCAGTCCCGGAGGG GTTTTAGAGCTAGAAATAGC
Cas9-MFE1-1	TTCGATTCCGGGTCGGCGCATGGTGAGACCCTGAAGGTTG GTTTTAGAGCTAGAAATAGC

Cas9-MFE1-2	TTCGATTCCGGGTCGGCGCAGGTGTTATCCCTTACATGGGG TTTTAGAGCTAGAAATAGC
Cas9-MFE1-3	TTCGATTCCGGGTCGGCGCACGTACTTCTGCTTAAGGAAGG TTTTAGAGCTAGAAATAGC
Cas9-MFE1-4	TTCGATTCCGGGTCGGCGCAGACAAGATCCCAGTCCTTGTG TTTTAGAGCTAGAAATAGC
Cas9-MFE1-5	TTCGATTCCGGGTCGGCGCAATACTTGAGCTCATTAGCCTG TTTTAGAGCTAGAAATAGC
Cas9-MFE1-6	TTCGATTCCGGGTCGGCGCACTGCTTTCGGAAGTAAGGCCG TTTTAGAGCTAGAAATAGC
Cas9-MFE1-7	TTCGATTCCGGGTCGGCGCAAAAGCAGGGTCGATGTGAAG GTTTTAGAGCTAGAAATAGC
Cas9-MFE1-8	TTCGATTCCGGGTCGGCGCAGTCGATGAAATTAAGGCCCTG TTTTAGAGCTAGAAATAGC
Cas9-MFE1-9	TTCGATTCCGGGTCGGCGCAGTTGTTGTCAACGATCTTGGG TTTTAGAGCTAGAAATAGC
Cas9-MFE1-10	TTCGATTCCGGGTCGGCGCACTTGGATCGGACAGACTCGA GTTTTAGAGCTAGAAATAGC
Cas12a-RAS2-1	TTTCTACTAAGTGTAGATGAGGCCCTAGATTACTTCAACGA CAAATTTCTACTAAGTGTA
Cas12a-RAS2-2	TTTCTACTAAGTGTAGATGACCACCTAACGACGCGAAAAA ACAAATTTCTACTAAGTGTA
Cas12a-RAS2-3	TTTCTACTAAGTGTAGATCGACATCACAGCCCCCCAGTCTT TGAATTTCTACTAAGTGTA
Cas12a-RAS2-4	TTTCTACTAAGTGTAGATGGCACCCGCACACCGGCCCCAGC TTAATTTCTACTAAGTGTA
Cas12a-RAS2-5	TTTCTACTAAGTGTAGATCATGAATCCGCATCCATGCTCGC GCAATTTCTACTAAGTGTA
Cas12a-RAS2-6	TTTCTACTAAGTGTAGATCATTGTCATTCTTGGAGAGGGAG GTAATTTCTACTAAGTGTA
Cas12a-RAS2-7	TTTCTACTAAGTGTAGATCGTCGCGACTGGGTGTGTCTGAT CGAATTTCTACTAAGTGTA
Cas12a-RAS2-8	TTTCTACTAAGTGTAGATGCGTCGTTAGGTGGTCCAAAACG AGAATTTCTACTAAGTGTA
Cas12a-RAS2-9	TTTCTACTAAGTGTAGATCTGAAGTTTCCATGAATCCGCAT CCAATTTCTACTAAGTGTA
Cas12a-RAS2-10	TTTCTACTAAGTGTAGATCGCGACTTTGCGCACTATAGATG AGAATTTCTACTAAGTGTA
Cas12a-MGA1-1	TTTCTACTAAGTGTAGATTGGGTGGTGGATTCGCTGAAGCG CTAATTTCTACTAAGTGTA
Cas12a-MGA1-2	TTTCTACTAAGTGTAGATATGGTCTGCGTCCAACGACTCGT TCAATTTCTACTAAGTGTA
Cas12a-MGA1-3	TTTCTACTAAGTGTAGATGGCGGCATGTGCTCGACCCGTTC TTAATTTCTACTAAGTGTA
Cas12a-MGA1-4	TTTCTACTAAGTGTAGATTGCGCCAGCTCAACATGTACGGC TTAATTTCTACTAAGTGTA

Cas12a-MGA1-5	TTTCTACTAAGTGTAGATGGTGGCCCATGGCGTGTGCCACC CGAATTTCTACTAAGTGTA
Cas12a-MGA1-6	TTTCTACTAAGTGTAGATTCAACAATCTGCAGCAGCGTCTG CAAATTTCTACTAAGTGTA
Cas12a-MGA1-7	TTTCTACTAAGTGTAGATTTGAACCCAGAAGGGGGCGACA AGAAATTTCTACTAAGTGTA
Cas12a-MGA1-8	TTTCTACTAAGTGTAGATGAGTGGTGCCGGGCTTCTTGTTA TCTTTTTTACGTCTAAGAA
Cas12a-MGA1-9	TTTCTACTAAGTGTAGATCCTGCTGGATGTCCTCCCGCGAA TCAATTTCTACTAAGTGTA
Cas12a-MGA1-10	TTTCTACTAAGTGTAGATGGCGCCGGAGGCTGTGTGGCGAC GGAATTTCTACTAAGTGTA
Cas12a-CAN1-1	TTTCTACTAAGTGTAGATCTACCCGATATCTGTCACAGTCG TTAATTTCTACTAAGTGTA
Cas12a-CAN1-2	TTTCTACTAAGTGTAGATACGACCCCAAGCTGACCGATGAC TCAATTTCTACTAAGTGTA
Cas12a-CAN1-3	TTTCTACTAAGTGTAGATGGCAGGAAACTCCAACGTCTACA TTAATTTCTACTAAGTGTA
Cas12a-CAN1-4	TTTCTACTAAGTGTAGATGTCTGCTGGCCTTCATGTCTGTGT CAATTTCTACTAAGTGTA
Cas12a-CAN1-5	TTTCTACTAAGTGTAGATGTGCCTCCATGGGCTGGCTATAC TGAATTTCTACTAAGTGTA
Cas12a-CAN1-6	TTTCTACTAAGTGTAGATCATCTTCTACATTGGCTCTATCTT CAATTTCTACTAAGTGTA
Cas12a-CAN1-7	TTTCTACTAAGTGTAGATTGGGGTTCTGGGCCTCACCGGCA GTAATTTCTACTAAGTGTA
Cas12a-CAN1-8	TTTCTACTAAGTGTAGATCTTGTGCGAGGGCACCTCCTCTG AGTTTTTTACGTCTAAGAA
Cas12a-CAN1-9	TTTCTACTAAGTGTAGATGTGCGGTTCCGGAGTCAGCCAGG GCAATTTCTACTAAGTGTA
Cas12a-CAN1-10	TTTCTACTAAGTGTAGATCTCGAATTTGCATCTTCTACATTG GAATTTCTACTAAGTGTA
Cas12a-MFE1-1	TTTCTACTAAGTGTAGATAGAGCCCCACCTACCCTAACGGC CCAATTTCTACTAAGTGTA
Cas12a-MFE1-2	TTTCTACTAAGTGTAGATGCCATGTAACCAGCACCGACCTC GTAATTTCTACTAAGTGTA
Cas12a-MFE1-3	TTTCTACTAAGTGTAGATGGGGGTGACACCCTTCTTGGTGT TGAATTTCTACTAAGTGTA
Cas12a-MFE1-4	TTTCTACTAAGTGTAGATGGTGCCTACAAGGTTACCCGAGC TGAATTTCTACTAAGTGTA
Cas12a-MFE1-5	TTTCTACTAAGTGTAGATATGTCCACCTCAACGGTACTTAC TCAATTTCTACTAAGTGTA
Cas12a-MFE1-6	TTTCTACTAAGTGTAGATCCGACTTTCTGGTGATTACAACC CTAATTTCTACTAAGTGTA
Cas12a-MFE1-7	TTTCTACTAAGTGTAGATCGGAACTTCGGCCAGACCAACT ACAATTTCTACTAAGTGTA

Cas12a-MFE1-8	TTTCTACTAAGTGTAGATGGTCGTTTCGCTTCGCTGCGCTTG TAATTTCTACTAAGTGTA
Cas12a-MFE1-9	TTTCTACTAAGTGTAGATAAGAAGTCAGCAGGGCCGTTAG GGTAATTTCTACTAAGTGTA
Cas12a-MFE1-10	TTTCTACTAAGTGTAGATTCCTTCTGTGTGGTGTCGTTTTGG GAATTTCTACTAAGTGTA

Supplementary Table 9. Transformation efficiencies measured as x10^6 transformants, for all replicates in the control and treatment strains.

Strain	***Replicate Transformation Efficiency (x10^6 transformants)***		
	**R1**	**R2**	**R3**
PO1f *Δku70*	689	621	543
PO1f Cas12a *Δku70*	506	429	441

Supplementary Table 10. Primers used for NGS fragment amplification

Primer name	Primer Sequence	Illumina Barcode (Reverse primer) / Pseudo-Barcode (Forward primer) for demultiplexing
ILU1-F	AATGATACGGCGACCACCGAGATCTACACTCTTTCCCTACACGACGCTCTTCCGATCTTTCCGGGTCGGCGCAAATTTC	^TTCCGG
ILU2-F	AATGATACGGCGACCACCGAGATCTACACTCTTTCCCTACACGACGCTCTTCCGATCTAGATCGGGTCGGCGCAAATTTCT	^AGATCG
ILU3-F	AATGATACGGCGACCACCGAGATCTACACTCTTTCCCTACACGACGCTCTTCCGATCTGCTATTCGGGTCGGCGCAAATTTCT	^GCTATT
ILU4-F	AATGATACGGCGACCACCGAGATCTACACTCTTTCCCTACACGACGCTCTTCCGATCTCAGGACTACGGGTCGGCGCAAATTTCT	^CAGGAC
ILU1-R	CAAGCAGAAGACGGCATACGAGATTCGCCTTGGTGACTGGAGTTCAGACGTGTGCTCTTCCGATCTTAGAGGATCTGGGCCTCGTGATAC	CAAGGCGA
ILU2-R	CAAGCAGAAGACGGCATACGAGATGACGAGAGGTGACTGGAGTTCAGACGTGTGCTCTTCCGATCTTAGAGGATCTGGGCCTCGTGATAC	CTCTCGTC
ILU3-R	CAAGCAGAAGACGGCATACGAGATAGACTTGGGTGACTGGAGTTCAGACGTGTGCTCTTCCGATCTTAGAGGATCTGGGCCTCGTGATAC	CCAAGTCT
ILU4-R	CAAGCAGAAGACGGCATACGAGATCTGTATTAGTGACTGGAGTTCAGACGTGTGCTCTTCCGATCTTAGAGGATCTGGGCCTCGTGATAC	TAATACAG
ILU5-R	CAAGCAGAAGACGGCATACGAGATCCTGAACCGTGACTGGAGTTCAGACGTGTGCTCTTCCGATCTTAGAGGATCTGGGCCTCGTGATAC	GGTTCAGG
ILU6-R	CAAGCAGAAGACGGCATACGAGATATCAGGTTGTGACTGGAGTTCAGACGTGTGCTCTTCCGATCTTAGAGGATCTGGGCCTCGTGATAC	AACCTGAT
ILU7-R	CAAGCAGAAGACGGCATACGAGATTAGGTGACGTGACTGGAGTTCAGACGTGTGCTCTTCCGATCTTAGAGGATCTGGGCCTCGTGATAC	GTCACCTA

ILU8-R	CAAGCAGAAGACGGCATACGAGATCGAACAGTGTGACTGGAGTTCAGACGTGTGCTCTTCCGATCTTAGAGGATCTGGGCCTCGTGATAC	ACTGTTCG
ILU9-R	CAAGCAGAAGACGGCATACGAGATGTTCGATCGTGACTGGAGTTCAGACGTGTGCTCTTCCGATCTTAGAGGATCTGGGCCTCGTGATAC	GATCGAAC
ILU10-R	CAAGCAGAAGACGGCATACGAGATACCTAGCTGTGACTGGAGTTCAGACGTGTGCCTTCCGATCTTAGAGGATCTGGGCCTCGTGATAC	AGCTAGGT
ILU11-R	CAAGCAGAAGACGGCATACGAGATAGAGATGAGTGACTGGAGTTCAGACGTGTGCTCTTCCGATCTTAGAGGATCTGGGCCTCGTGATAC	TCATCTCT
ILU12-R	CAAGCAGAAGACGGCATACGAGATCTGGACTTGTGACTGGAGTTCAGACGTGTGCTCTTCCGATCTTAGAGGATCTGGGCCTCGTGATAC	AAGTCCAG

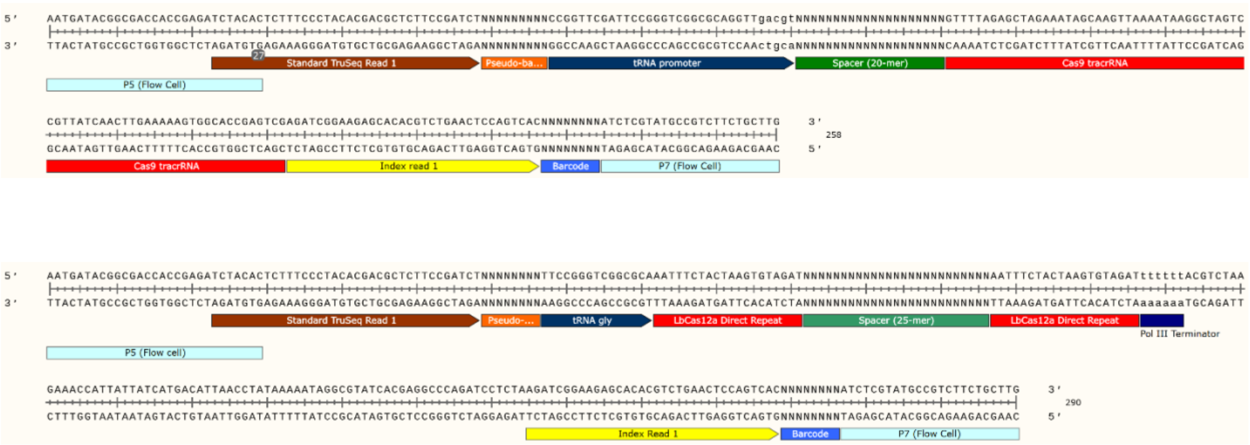

Supplementary Figure 8. Schematic and sequence information of Cas9 (top) and Cas12a (bottom) amplicons for NGS. Amplicons contain (i) P5 and P7 sequences (light blue) that are necessary for binding with the flow cell in Illumina sequencers, (ii) TruSeq adapter (brown) for binding of the sequencing primer, (iii) a portion of $tRNA^{gly}$ (black) expressing the sgRNA, (iv) Cas9 or Cas12 spacer (green) (v) Cas12a associated direct repeats or a portion of the Cas9 tracrRNA sequence (red), (vi) Universal 8 bp Illumina barcodes (blue), (vii) Index read 1 sequence for the binding of primers to sequence the Illumina barcodes, and (viii) 4-9 nt pseudo-barcodes (orange) at the 5' end between the TruSeq and $tRNA^{gly}$ which help demultiplex replicates that contain the same illumine barcode.

Supplementary Table 11. Parameters for bioinformatics tools used in analysis of NGS reads

Tool	Version	Parameters*
FastQC	v0.11.8	Default settings
Cutadapt	Galaxy Version 1.16.6 [8]	The 3 biological replicates of a given sample at a given time-point always had the same reverse primer containing the Illumina barcode, and forward primers ILU1-F, ILU3-F and ILU4-F; or ILU2-F, ILU3-F and ILU4-F each containing different pseudo-barcodes. Thus Cutadapt was used to demultiplex biological replicates from each other.<br>▪ 5’ (Front) anchored 6 bp pseudo-barcodes to be demultiplexed (-g): ^NNNNNN (refer to previous table for pseudo-barcode-forward primer association).<br>▪ Maximum error rate (--error-rate): 0.2<br>▪ Match times (--times): 1<br>▪ Minimum overlap length (--overlap): 4<br>▪ Multiple output: Yes (Each demultiplexed readset is written to a separate file)
Trimmomatic	v0.38	▪ HEADCROP: 29 (if amplified by ILU1-F); or 31 (if amplified by ILU2-F); or 32 (if amplified by ILU3-F); or 34 (if amplified by ILU4-F)<br>▪ CROP: 25
Bowtie2	v2.4.2	▪ Number of allowed mismatches in seed alignment (-N): 1<br>▪ Length of the seed substring (-L): 21<br>▪ Function governing interval between seed substrings in multiseed alignment (-i): S,1,0.50<br>▪ Function governing maximum number of ambiguous characters (--n-ceil): L,0,0.15<br>▪ Alignment mode: end-to-end<br>▪ Number of attempts of consecutive seed extension events (-D): 20<br>▪ Number of times re-seeding occurs for repetitive reads: 3<br>▪ Save mapping statistics: Yes

Note: All parameters other than those mentioned here are kept at default values.

Supplementary Table 12. Correlation of SRA files names to demultiplexing information

SRA file name	SRA sample name	Demultiplexing needed	Pseudo-Barcode for Demultiplexing with CutAdapt**	Readsets contained
**GW-Cpf1_Control-2_S2_R1_001.fastq.gz**	PO1f_dku70_day2_All3reps	Yes	^AGATCG	Replicate #1
			^GCTATT	Replicate #2
			^CAGGAC	Replicate #3
**GW-Cpf1_Control-4_S4_R1_001.fastq.gz**	PO1f_dku70_day4_All3reps	Yes	^AGATCG	Replicate #1
			^GCTATT	Replicate #2
			^CAGGAC	Replicate #3
**GW-Cpf1_Control-6_S6_R1_001.fastq.gz**	PO1f_dku70_day6_All3reps	Yes	^AGATCG	Replicate #1
			^GCTATT	Replicate #2
			^CAGGAC	Replicate #3

**Yl-Cpf1_CS-2_S2_R1_001.fastq.gz**	PO1f_LbCas12a_dku70_day2_All3reps	Yes	^AGATCG	Replicate #1
			^GCTATT	Replicate #2
			^CAGGAC	Replicate #3
**Yl-Cpf1_CS-4_S4_R1_001.fastq.gz**	PO1f_LbCas12a_dku70_day4_All3reps	Yes	^AGATCG	Replicate #1
			^GCTATT	Replicate #2
			^CAGGAC	Replicate #3
**Yl-Cpf1_CS-6_S6_R1_001.fastq.gz**	PO1f_LbCas12a_dku70_day6_All3reps	Yes	^AGATCG	Replicate #1
			^GCTATT	Replicate #2
			^CAGGAC	Replicate #3
**GW_Yl_Cpf1-7_S7_R1_001.fastq.gz**	LbCas12a_Library_Rep1	No	N/A	Replicate #1

**GW_Yl_Cpf1-8_S8_R1_001.fastq.gz**	LbCas12a_Library_Rep2	No	N/A	Replicate #2
**GW_Yl_Cpf1-9_S9_R1_001.fastq.gz**	LbCas12a_Library_Rep3	No	N/A	Replicate #3

** The symbol '^' before the barcode sequence represents that it is anchored, i.e the read begins with the barcode sequence from the 5' end. This information is needed for demultiplexing with CutAdapt.

References

1. Schwartz, C. *et al.* Validating genome-wide CRISPR-Cas9 function improves screening in the oleaginous yeast Yarrowia lipolytica. *Metab. Eng.* **55**, 102–110 (2019).
2. Guo, J. *et al.* Improved sgRNA design in bacteria via genome-wide activity profiling. *Nucleic Acids Res.* **46**, 7052–7069 (2018).
3. Wang, D. *et al.* Optimized CRISPR guide RNA design for two high-fidelity Cas9 variants by deep learning. *Nat. Commun.* **10**, 4284 (2019).
4. Moreb, E. A. & Lynch, M. D. Genome dependent Cas9/gRNA search time underlies sequence dependent gRNA activity. *Nat. Commun.* **12**, 5034 (2021).
5. Kim, H. K. *et al.* Deep learning improves prediction of CRISPR-Cpf1 guide RNA activity. *Nat. Biotechnol.* **36**, 239–241 (2018).
6. Ramesh, A., Ong, T., Garcia, J. A., Adams, J. & Wheeldon, I. Guide RNA Engineering Enables Dual Purpose CRISPR-Cpf1 for Simultaneous Gene Editing and Gene Regulation in Yarrowia lipolytica. *ACS Synthetic Biology* vol. 9 967–971 (2020).
7. Schwartz, C. M., Hussain, M. S., Blenner, M. & Wheeldon, I. Synthetic RNA Polymerase III Promoters Facilitate High-Efficiency CRISPR-Cas9-Mediated Genome Editing in Yarrowia lipolytica. *ACS Synth. Biol.* **5**, 356–359 (2016).
8. Jalili, V. *et al.* The Galaxy platform for accessible, reproducible and collaborative biomedical analyses: 2020 update. *Nucleic Acids Res.* **48**, W395–W402 (2020).
