# Supplementary material for: Genome-wide functional screens enable the prediction of high activity CRISPR-Cas9 and -Cas12a guides in Yarrowia lipolytica
Source: Nat Commun. 2022 Feb 17;13:922. doi: 10.1038/s41467-022-28540-0 (PMC8854577; doi:10.1038/s41467-022-28540-0)
Supplement: Supplementary file 4 — Description of Additional Supplementary Files [file 41467_2022_28540_MOESM4_ESM.pdf]

**Title: Supplementary Data 1.**
**Description:** Cutting scores of all Cas9 sgRNAs (including nontargeting guides) at days 2, 4 and 6.

**Title: Supplementary Data 2.**
**Description:** Cutting scores of all Cas12a sgRNAs (including nontargeting guides) at days 2, 4 and 6.

**Title: Supplementary Data 3.**
**Description:** Normalized sgRNA abundance for both the control strain (not expressing Cas9) and treatment strain (expressing Cas9 and disrupted for KU70) at days 2, 4 and 6, when transformed with the Cas9 library.

**Title: Supplementary Data 4.**
**Description:** Normalized sgRNA abundance for both the control strain (not expressing Cas12a) and treatment strain (expressing Cas12a and disrupted for KU70) at days 2, 4 and 6, when transformed with the Cas12a library.

**Title: Supplementary Data 5.**
**Description:** Average normalized nucleosome occupancy score for every sgRNA in the Cas9 and Cas12a libraries for which MNase-Seq data was available.
